# Supplementary material for: Camellia Seed Cake Extract Supports Hair Growth by Abrogating the Effect of Dihydrotestosterone in Cultured Human Dermal Papilla Cells
Source: Molecules. 2022 Sep 29;27(19):6443. doi: 10.3390/molecules27196443 (PMC9572183; doi:10.3390/molecules27196443)
Supplement: Supplementary file 1 [file molecules-27-06443-s001.zip › molecules-1911701-supplementary.pdf]

Supplementary Materials

Camellia Seed Cake Extract Supports Hair Growth by Abrogating the Effect of Dihydrotestosterone in Cultured Human Dermal Papilla Cells

Ling Ma [1,*], Huchi Shen [2], Chengge Fang [2], Timson Chen [1] and Jing Wang [1,*]

[1] Adolph Innovation Laboratory, Guangzhou Degu Personal Care Products Co., Ltd., Guangzhou 510000, China

[2] Key Laboratory of Synthetic and Biological Colloids, Ministry of Education, School of Chemical and Material Engineering, Jiangnan University, Wuxi 214122, China

* Correspondence: maling@adolph.cn (L.M.); jingwang@jiangnan.edu.cn (J.W.)

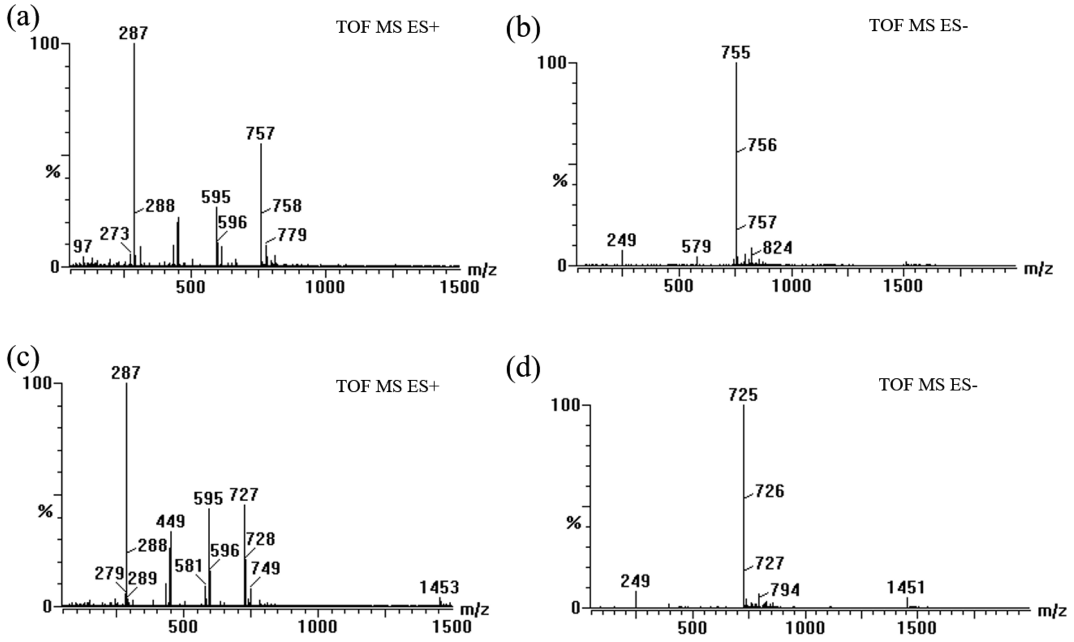

Figure S1. Mass spectrum of compound 1(a, b) and compound 2 (c, d)
